# Long-term safety of secukinumab in patients with moderate-to-severe plaque psoriasis, psoriatic arthritis, and ankylosing spondylitis: integrated pooled clinical trial and post-marketing surveillance data

**DOI:** 10.1186/s13075-019-1882-2

**Published:** 2019-05-02

**Authors:** A. Deodhar, P. J. Mease, I. B. McInnes, X. Baraliakos, K. Reich, A. Blauvelt, C. Leonardi, B. Porter, A. Das Gupta, A. Widmer, L. Pricop, T. Fox

**Affiliations:** 1 Division of Arthritis & Rheumatic Diseases (OP-09), Oregon Health & Science University, 3181 SW Sam Jackson Park Road, Portland, OR 97239-3098 USA; 2 Swedish Medical Center and University of Washington, Seattle, USA; 3 University of Glasgow, Glasgow, UK; 4 Rheumazentrum Ruhrgebiet Herne, Ruhr-University Bochum, Bochum, Germany; 5 Translational Research in Inflammatory Skin Diseases, Institute for Health Services Research in Dermatology and Nursing, University Medical Center Hamburg-Eppendorf, Skinflammation® Center, Hamburg, Germany; 6 Dermatologikum Berlin, Berlinermatologikum Berlin and SCIderm Research Institute, Hamburg, Germany; 7 Oregon Medical Research Center, Portland, USA; 8 Saint Louis University Health Science Center, St. Louis, USA; 9 Novartis Pharmaceuticals Corporation, East Hanover, USA; 10 Novartis Healthcare Private Limited, Hyderabad, India; 11 Novartis Pharma AG, Basel, Switzerland

**Keywords:** Spondyloarthritis, Safety, Biologics, Interleukin, TNF inhibitors

## Abstract

**Background:**

Secukinumab, a fully human immunoglobulin G1-kappa monoclonal antibody that directly inhibits interleukin (IL)-17A, has been shown to have robust efficacy in the treatment of moderate-to-severe psoriasis (PsO), psoriatic arthritis (PsA), and ankylosing spondylitis (AS) demonstrating a rapid onset of action and sustained long-term clinical responses with a consistently favorable safety profile in multiple Phase 2 and 3 trials. Here, we report longer-term pooled safety and tolerability data for secukinumab across three indications (up to 5 years of treatment in PsO and PsA; up to 4 years in AS).

**Methods:**

The integrated clinical trial safety dataset included data pooled from 21 randomized controlled clinical trials of secukinumab 300 or 150 or 75 mg in PsO (14 Phase 3 trials and 1 Phase 4 trial), PsA (3 Phase 3 trials), and AS (3 Phase 3 trials), along with post-marketing safety surveillance data with a cut-off date of June 25, 2017. Adverse events (AEs) were reported as exposure-adjusted incident rates (EAIRs) per 100 patient-years. Analyses included all patients who received ≥ 1 dose of secukinumab.

**Results:**

A total of 5181, 1380, and 794 patients from PsO, PsA, and AS clinical trials representing secukinumab exposures of 10,416.9, 3866.9, and 1943.1 patient-years, respectively, and post-marketing data from patients with a cumulative exposure to secukinumab of ~ 96,054 patient-years were included in the analysis. The most frequent AE was upper respiratory tract infection. EAIRs across PsO, PsA, and AS indications were generally low for serious infections (1.4, 1.9, and 1.2, respectively), *Candida* infections (2.2, 1.5, and 0.7, respectively), inflammatory bowel disease (0.01, 0.05, and 0.1, respectively), and major adverse cardiac events (0.3, 0.4, and 0.6, respectively). No cases of tuberculosis reactivation were reported. The incidence of treatment-emergent anti-drug antibodies was low with secukinumab across all studies, with no discernible loss of efficacy, unexpected alterations in pharmacokinetics, or association with immunogenicity-related AEs.

**Conclusions:**

Secukinumab demonstrated a favorable safety profile over long-term treatment in patients with PsO, PsA, and AS. This comprehensive assessment demonstrated that the safety profile of secukinumab was consistent with previous reports in patients with PsO, PsA, and AS, supporting its long-term use in these chronic conditions.

**Electronic supplementary material:**

The online version of this article (10.1186/s13075-019-1882-2) contains supplementary material, which is available to authorized users.

## Article summary

### Strengths (S) and limitations (L) of this study

- (S) Integrates safety data from a large patient population pooled over 21 clinical trials across multiple indications complemented with large post-marketing surveillance safety data
- (S) Exposure-adjusted incidence rates for reporting safety data enhances the robustness of the results by adjusting for treatment duration
- (S) Provides valuable evidence on the comprehensive safety profile of secukinumab that should inform clinical decision-making
- (L) Conduct of clinical trials is protocol-specified and may not fully reflect real-world clinical experience and lack of a long-term placebo comparison, due to ethical considerations, limits comparisons
- (L) Inflammatory bowel disease events were not adjudicated and post-marketing safety surveillance results have not been separated by individual dose regimen or by indication

## Background

Interleukin (IL)-17A is involved in mucocutaneous defense [1] and plays a critical role in the pathogenesis of a range of immune-mediated diseases, including psoriasis (PsO), psoriatic arthritis (PsA), and ankylosing spondylitis (AS) [2–4]. The disease pathogenesis of PsO, PsA, and AS is complex and involves an interplay among environmental, genetic, and immune triggers. There is a considerable genetic overlap with other immune-mediated diseases, and there is often evidence of common immune dysregulation, making these patients more susceptible to infections and/or adverse events (AEs) compared with the general population [5–8]. Furthermore, introduction of a foreign protein product, such as a monoclonal antibody, into the human body carries the possibility of an immunologic response and formation of anti-drug antibodies (ADAs) that may impact treatment efficacy and safety [9]. The presence of co-morbidities and use of concomitant medications further impacts these safety risks.

Biologic agents used in the management of PsO or PsA or AS have immunomodulatory potential via their effects on Type 17 (T helper [Th] and T Cell17) cell cytokines and other pathways, emphasizing the need to understand the unique safety profile of individual agents [10]. IL-17A also plays a role in the defense against extracellular pathogens [10] and other immune mechanisms. Therapy for these chronic diseases is typically intended for long-term use and, hence, understanding the long-term efficacy and safety of therapeutic compounds is particularly relevant for clinical decision-making.

Fully human monoclonal antibodies are derived from human gene sequences alone and are generally considered to have the least immunogenic potential among biologic agents [11]. Secukinumab, a fully human immunoglobulin G1-kappa monoclonal antibody that directly inhibits IL-17A, has been shown to have robust efficacy in the treatment of moderate-to-severe PsO [12–15], PsA [16–18], and AS [19, 20] demonstrating a rapid onset of action and sustained long-term clinical responses with a consistently favorable safety profile observed in the context of multiple Phase 2 and 3 trials [17, 20, 21]. Secukinumab is currently approved in > 80 countries for use in PsO/PsA/AS with over 150,000 patients treated [22].

Here, we present integrated pooled clinical trial safety data for secukinumab following long-term exposure of up to 5 years in PsO and PsA and up to 4 years in AS. Year-by-year AE rates in this cohort and the post-marketing data available from the secukinumab periodic safety update report (PSUR) submitted to global health authorities with a reporting cut-off date of June 25, 2017, are also reported and reviewed.

## Methods

### Studies and patients

**Fig. 1:**
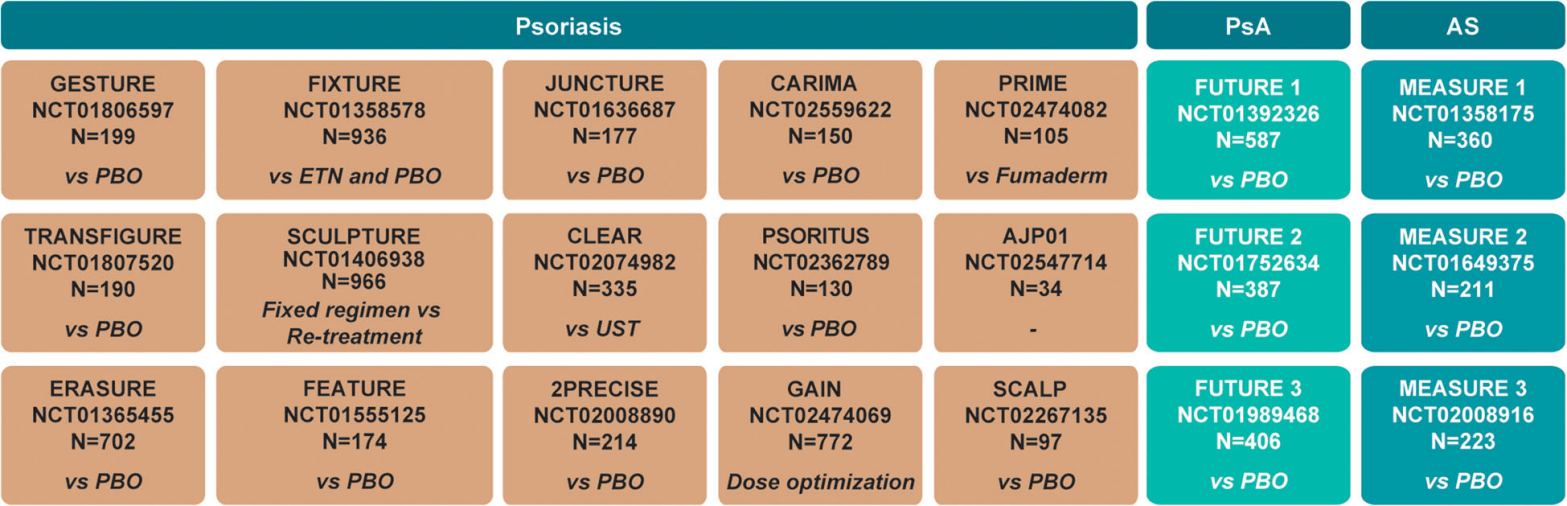
Studies included in the pooled analysis. *ETN* etanercept, *PBO* placebo, *UST* ustekinumab

The integrated clinical trial safety dataset included data pooled from 21 randomized controlled clinical trials of secukinumab in PsO (14 Phase 3 trials and 1 Phase 4 trial; *N* = 5181), PsA (3 Phase 3 trials; *N* = 1380), and AS (3 Phase 3 trials; *N* = 794) indications (see Fig. 1 for details), along with post-marketing surveillance data for secukinumab across the PsO, PsA, and AS indications from December 26, 2014 to June 25, 2017. Multiple secukinumab dose regimens were used in these studies and included intravenous (up to 10 mg/kg) or subcutaneous (s.c.; 75, 150, or 300 mg) loading followed by s.c. maintenance dosing (75, 150, or 300 mg). Placebo-treated patients were re-randomized to secukinumab between 12 and 24 weeks’ post-baseline in the various studies. Eligible patients aged ≥ 18 years with moderate-to-severe plaque PsO or active PsA or AS were enrolled based on pre-specified eligibility criteria, which, along with the design of each study, have been reported in detail elsewhere [12, 15, 16, 18, 20, 23–26]. Of note, patients enrolled in these trials could have active/ongoing cardiovascular disease (unless severe or uncontrolled), previous history of inflammatory bowel disease (IBD), including Crohn’s disease (CD) or uveitis (but not if active and ongoing), history of basal cell carcinoma or actinic keratosis (successfully treated with no evidence of recurrence in the past 3 months), carcinoma in situ of the cervix or non-invasive malignant colon polyps (successfully removed), or latent tuberculosis (TB; prophylactic treatment started prior to enrollment). Patients could continue the following medications at stable doses: sulfasalazine, methotrexate, corticosteroids, and non-steroidal anti-inflammatory drugs. Patients with an inadequate response (if taken ≥ 3 months) or intolerance to tumor necrosis factor (TNF) inhibitors (not more than one in AS studies and not more than 3 in PsO and PsA studies) could also be included. Two studies (ERASURE and FIXTURE) in the PsO pool also allowed patients with a prior history of usage of biologics other than TNF inhibitors (alefacept, briakinumab, efalizumab, and ustekinumab) after an appropriate washout period.

### Safety assessments

Safety analyses included all patients who had received ≥ 1 dose of study medication, with data pooled at the individual patient level for all secukinumab treatment groups (any secukinumab) separately for the studies by indication. Analysis of the entire secukinumab treatment period was performed on safety data pooled at the patient level from the date of treatment initiation up to June 25, 2017. Analysis of the safety data was also performed by the approved secukinumab 300 and 150 mg doses separately for the studies by indication. Risks for AEs, serious AEs (SAEs), and selected AEs were expressed as exposure-adjusted incidence rates (EAIRs) per 100 patient-years for the entire treatment period. The EAIR was defined as the number of subjects exposed to the drug and experiencing a certain event divided by the total exposure time of all subjects who were at risk for the event.

AEs were coded using the Medical Dictionary for Regulatory Activities (MedDRA) version 20.0 preferred terms (PTs) (https://www.meddra.org/). ADAs were measured using a Meso-scale Discovery bridging assay [27]. ADA-positive samples were analyzed to week 52 for drug-neutralizing potential, immunogenicity-related AEs, and impact on pharmacokinetics (PK) and efficacy of secukinumab. All clinical studies were conducted in compliance with the Declaration of Helsinki [28], International Council for Harmonization Guidelines for Good Clinical Practice, and local country regulations.

## Results

The results are presented here in two sections: pooled clinical trial safety results and results from the post-marketing safety surveillance. Unless specified otherwise, the results predominantly reflect the pooled clinical trial data.

### Pooled clinical trial safety results

#### Baseline characteristics

Pooled safety analyses included 7355 secukinumab-treated patients with an overall exposure of 16,226.9 patient-years. A total of 5181 patients (representing 10,416.9 patient-years of exposure) were included in the PsO pooled analyses, 1380 patients (representing 3866.9 patient-years of exposure) in the PsA pooled analyses, and 794 patients (representing 1943.1 patient-years of exposure) in the AS pooled analyses. Demographics and baseline disease characteristics of the pooled secukinumab groups are provided in Table 1. The baseline rates of hypertension (39.9%), hyperlipidemia (23.0%), and diabetes (12.4%) were comparatively higher in the PsA pool than in the other two indications. The mean body mass index was higher in the PsO (29.1) and PsA (29.8) pools compared with the AS pool (27.2). The baseline rate of uveitis was higher in the AS pool (17%), which was expected given the propensity of this patient population to uveitis. A history of IBD (unspecified IBD, CD, and/or ulcerative colitis [UC]) was reported in 15 (0.3%) PsO, 8 (0.6%) PsA, and 25 (3.1%) AS patients, respectively.

**Table 1:** Baseline demographics and disease characteristics of pooled clinical trial patient population

Characteristic	PsO studies	PsA studies	AS studies
	Any secukinumab *N* = 5181	Any secukinumab *N* = 1380	Any secukinumab *N* = 794
Age (years), mean (SD)	45.7 (13.3)	48.8 (12.0)	42.4 (12.3)
Female, *n* (%)	1743 (33.6)	742 (53.8)	265 (33.4)
Caucasian, *n* (%)	4236 (81.8)	1212 (87.8)	612 (77.1)
BMI, mean (SD)	29.1 (6.6)	29.8 (6.3)	27.2 (5.5)
Relevant medical history or current medical condition, *n* (%)			
Hypertension	1089 (21.0)	551 (39.9)	176 (22.2)
Hyperlipidemia	667 (12.9)	318 (23.0)	65 (8.2)
Diabetes mellitus	341 (6.6)	171 (12.4)	22 (2.8)
IBD	0 (0)	4 (0.3)	17 (2.1)
Crohn’s disease	5 (0.1)	2 (0.1)	5 (0.6)
Ulcerative colitis	10 (0.2)	2 (0.1)	3 (0.4)
Uveitis	0 (0)	8 (0.6)	135 (17.0)
Current smoker	1585 (30.6)	262 (19.0)	234 (29.5)
Anti-TNF inadequate responder	784 (15.1)	435 (31.5)	227 (28.6)

*AS* ankylosing spondylitis, *BMI* body mass index, *IBD* inflammatory bowel disease, *N* number of patients in the analysis, *n* number of patients with a response, *PsA* psoriatic arthritis, *PsO* psoriasis, *SD* standard deviation, *TNF* tumor necrosis factor

### Safety summary

The EAIRs of any AE with secukinumab treatment across the entire safety period were 204.4, 147.0, and 140.1 per 100 patient-years in the PsO, PsA, and AS pools, respectively. The EAIRs of any SAE with secukinumab treatment across the entire safety period were 6.9, 7.9, and 6.3 per 100 patient-years in the PsO, PsA, and AS pools, respectively, showing no discernible pattern across the treatment groups. A total of 9 (0.2%) deaths were reported in the secukinumab-treated PsO cohort (the primary reasons for death being pulmonary embolism, arrhythmia, alcohol poisoning, ruptured aneurysm, and myocardial infarction [2 patients] in the secukinumab 300 mg group and cerebral hemorrhage, cardiorespiratory arrest, and completed suicide in the secukinumab 150 mg group). There were 11 (0.8%) deaths in the PsA pool (150 mg group: acute myocardial infarction, septic shock, sepsis, pneumonia, metastatic small cell lung cancer, pancreatic carcinoma, and cardiac failure [2 patients]; 75 mg group: squamous cell carcinoma of the pharynx, myocardial infarction, and cerebrovascular event) and 5 (0.6%) in the AS pool (150 mg group: 1 unknown; 75 mg group: acute respiratory failure, cerebrovascular accident, acute myocardial infarction, and respiratory arrest). Discontinuations due to AEs numbered 331 (6.4%), 104 (7.5%), and 58 (7.3%) in the PsO, PsA, and AS pools, respectively (Table 2). The EAIRs of selected AEs with secukinumab were comparable across the PsO, PsA, and AS studies (Table 3). The rates were comparable with those reported previously [15, 16, 18, 20], and no new safety signals were identified from those reported previously.

**Table 2:** Summary of pooled safety data from secukinumab clinical trials

	PsO studies	PsA studies	AS studies
	Any secukinumab *N* = 5181	Any secukinumab *N* = 1380	Any secukinumab *N* = 794
Total exposure, pt-years	10,416.9	3866.9	1943.1
Min–max exposure (days)	1–1825	8–1827	1–1530
Death, *n* (%)	9 (0.2)	11 (0.8)	5 (0.6)
Discontinuations due to AEs, *n* (%)	331 (6.4)	104 (7.5)	58 (7.3)
AEs, EAIR per 100 pt-years (95% CI)			
Any AE	204.4 (198.4, 210.5)	147.0 (138.9, 155.5)	140.1 (129.8, 151.0)
Any serious AE	6.9 (6.3, 7.4)	7.9 (7.0, 8.9)	6.3 (5.2, 7.6)
Most common AEs^1^			
Viral URTI^2^	21.0 (19.9, 22.0)	12.1 (10.9, 13.4)	9.8 (8.4, 11.5)
Headache	6.2 (5.8, 6.8)	3.8 (3.2, 4.5)	5.3 (4.3, 6.5)
Diarrhea	3.8 (3.4, 4.2)	3.7 (3.1, 4.4)	5.2 (4.2, 6.4)
URTI	5.4 (4.9, 5.9)	9.1 (8.1, 10.2)	5.2 (4.2, 6.4)

^1^AEs in the secukinumab group that occurred with an IR > 5.0 during the entire safety period in any of the pooled groups

^2^Includes cases of common cold (LLT)

*AE* adverse event, *AS* ankylosing spondylitis, *CI* confidence interval, *EAIR* exposure-adjusted incidence rate per 100 patient-years, *IR* incidence rate, *LLT* low-level term, *N* number of patients in the analysis, *n* number of patients with a response, *PsA* psoriatic arthritis, *PsO* psoriasis, *pt* patient, *URTI* upper respiratory tract infection

**Table 3:** Selected AEs with secukinumab across pooled clinical trials

Variable	PsO studies	PsA studies	AS studies
	Any secukinumab *N* = 5181	Any secukinumab *N* = 1380	Any secukinumab *N* = 794
	EAIR per 100 patient-years (95% CI)		
Serious infections^1^	1.4 (1.2, 1.6)	1.9 (1.5, 2.4)	1.2 (0.8, 1.8)
*Candida* infections^2^	2.2 (1.9, 2.5)	1.5 (1.1, 2.0)	0.7 (0.4, 1.2)
IBD^3^	0.01 (0.00, 0.05)	0.05 (0.01, 0.2)	0.1 (0.0, 0.3)
Crohn’s disease^3^	0.05 (0.02, 0.1)	0.08 (0.02, 0.2)	0.4 (0.2, 0.8)
Ulcerative colitis^3^	0.1 (0.07, 0.2)	0.08 (0.02, 0.2)	0.2 (0.1, 0.5)
MACE^4^	0.3 (0.2, 0.5)	0.4 (0.3, 0.7)	0.6 (0.3, 1.1)
Neutropenia^3^	0.3 (0.2, 0.4)	0.2 (0.1, 0.4)	0.5 (0.3, 1.0)
Uveitis^3^	0.02 (0.0, 0.07)	0.1 (0.0, 0.2)	1.4 (0.9, 2.0)
Malignancy^5^	0.8 (0.6, 1.0)	1.1 (0.8, 1.5)	0.5 (0.2, 0.9)

Approximation was not done if EAIR is less than 0.1

^1^Values are based on system organ class: infections and infestations

^2^Values are based on the high-level term

^3^Values are based on the preferred term

^4^Values are based on Novartis MedDRA query, which comprises (1) any MI, (2) any CVA, and (3) all other CV events that are fatal, out of a listing of 2200+ terms

^5^Values are based on standardized MedDRA query

*AE* adverse event, *AS* ankylosing spondylitis, *CI* confidence interval, *CV* cardiovascular, *CVA* CV accident, *EAIR* exposure-adjusted incidence rate per 100 patient-years, *IBD* inflammatory bowel disease, *MACE* major adverse cardiovascular event, *MedDRA* Medical Dictionary for Regulatory Activities, *MI* myocardial infarction, *N* number of patients in the analysis, *n* number of patients with a response, *PsA* psoriatic arthritis, *PsO* psoriasis

### Infections

Over the entire safety period, upper respiratory tract infection (URTI) was the most common type of infection across all indications. In an overall dataset of 7355 patients, the EAIRs for serious infections were 1.4, 1.9, and 1.2 per 100 patient-years in the PsO, PsA, and AS pools, respectively (Table 3). A total of 14 cases (EAIR of 0.13 per 100 patient-years) of opportunistic infections were reported in the PsO pool, which included 10 cases of esophageal candidiasis, 3 cases of gastrointestinal candidiasis, and 1 case of herpes zoster infection. Eight cases (EAIR of 0.21 per 100 patient-years) of opportunistic infections were reported in the PsA pool, which included 4 cases of esophageal candidiasis and 1 case each of herpes zoster infection, toxoplasmosis, *Mycobacterium avium* complex infection, and pneumonia. The 2 cases (EAIR of 0.1 per 100 patient-years) of opportunistic infections reported in the AS pool were herpes zoster infection and esophageal candidiasis. A total of 5 cases (EAIR of 0.05 per 100 patient-years) of sepsis (PT) were reported in the PsO pool, 3 (EAIR of 0.08 per 100 patient-years) in the PsA pool, and none in the AS pool. Most infections were mild to moderate.

### *Candida* infection

Cutaneous or mucosal *Candida* infection (MedDRA high-level term) was reported in 221 (EAIR of 2.2 per 100 patient-years) patients across the PsO studies, 57 (EAIR of 1.5 per 100 patient-years) patients across the PsA studies, and 13 (EAIR of 0.7 per 100 patient-years) patients across the AS studies (Table 3 and Additional file 1: Table S1). All cases of *Candida* infection were localized, most were mild or moderate in severity (except for 4 cases in the PsO pool that were considered severe), were self-limited or responsive to standard treatment, and did not lead to discontinuation of study treatment. No cases of systemic candidiasis were reported in patients treated with secukinumab across all studies.

### Neutropenia

Over the entire treatment period, the EAIRs per 100 patient-years for neutropenia with secukinumab treatment were 0.3, 0.2, and 0.5 in the PsO, PsA, and AS studies, respectively (Table 3). In the PsO pool, grade 3 neutropenia (defined as an absolute neutrophil count between 1.0 and 0.5 × 10^9^/L) was reported in 33 (0.6%) patients and grade 4 neutropenia (defined as an absolute neutrophil count of less than 0.5 × 10^9^/L) was reported in 2 (0.04%) patients. Of the cases of neutropenia, 7 were reported as severe, while the remaining were mild or moderate in severity (viral URTI was the most frequently co-reported AE [17 cases]); there were 5 cases in which study treatment was withdrawn. In the PsA pool, 12 (0.9%) patients reported grade 3, and 3 (0.2%) patients reported grade 4 neutropenia. All cases were either mild or moderate in severity (anemia, diarrhea, fatigue, leukopenia, URTI, and urinary tract infection were the most frequently co-reported AEs [3 cases each]), with 1 instance in which study treatment was withdrawn. In the AS pool, 9 (1.1%) patients reported grade 3, and 5 (0.6%) patients reported grade 4 neutropenia in the AS pool. All cases of neutropenia reported in the AS pool were either mild or moderate in severity (diarrhea was the most frequently co-reported AE [4 cases]), with none leading to study treatment discontinuation.

### Inflammatory bowel disease

The EAIRs per 100 patient-year exposure for CD, UC, or unspecified IBD combined ranged from 0.01 to 0.1 in PsO, 0.05 to 0.08 in PsA, and 0.1 to 0.4 in the AS pools (Table 3). There were 41 (0.6%) reported cases of IBD (including CD and UC). Of these, 30 (0.4%) were new-onset cases. Across indications, 14 (0.2%) patients who reported IBD discontinued the study.

### Major adverse cardiovascular events

The EAIR per 100 patient-years for major adverse cardiovascular events (MACE) with secukinumab treatment over the entire treatment period was 0.3, 0.4, and 0.6 in the PsO, PsA, and AS pools, respectively (Table 3).

### Uveitis

In the PsO pool, the EAIR for uveitis was 0.02 per 100 patient-years over the entire treatment period; all cases of uveitis (*n* = 2) were de novo. In the PsA pool, the EAIR for uveitis was 0.1 per 100 patient-years over the entire treatment period; all cases of uveitis (*n* = 3) were de novo. The EAIR for uveitis in the AS pool was 1.4 per 100 patient-years over the entire treatment period; a total of 12 (46%) cases were de novo (Table 3). Among all cases of uveitis (*n* = 26) in the AS pool, one was reported as severe and the remainder were reported as mild or moderate. A total of 135 (17%) patients in the AS pool reported pre-existing (but not active or ongoing) uveitis at baseline, and 589 (74.2%) were HLA-B27 positive, which has a known association with the development of uveitis.

### Malignancy

Malignant or unspecified tumors (Standardized MedDRA Queries [SMQ]) were reported in 81 patients (EAIR of 0.8 per 100 patient-years) in the PsO pool, which included 76 patients with non-hematological malignant tumors, 2 patients each with a hematological malignant tumor or a non-hematological tumor of unspecified malignancy, and 1 patient with a hematological tumor of unspecified malignancy. In the PsA pool, malignant or unspecified tumors were reported in 43 patients (EAIR of 1.1 per 100 patient-years), which included 37 patients with non-hematological malignant tumors, 5 patients with a non-hematological tumor of unspecified malignancy, and 1 patient with a hematological malignant tumor. In the AS pool, malignant or unspecified tumors were reported in 9 patients (EAIR of 0.5 per 100 patient-years), which included 6 patients with non-hematological malignant tumors, 2 patients with a non-hematological tumor of unspecified malignancy, and 1 patient with a hematological malignant tumor.

### Suicidality

Over the entire treatment period, in the PsO studies, 8 patients (0.2%) reported some form of suicidality-related AEs, including 4 attempted suicides, 1 completed suicide, 2 cases of suicidal ideation, and 1 case of suicidal depression. Three cases (0.2%) of suicidal ideation were reported in the PsA studies. All suicidality cases presented at least one of the following: depression, anxiety, insomnia, bipolar disorder, co-medication with psychoactive drugs, alcoholism, and/or ongoing psychological or socio-economic issues. No cases of suicidality-related AEs were reported in the AS studies on secukinumab treatment. These suicidality safety results with secukinumab were consistent with an earlier report from a pooled analysis of data from 10 clinical studies in moderate-to-severe plaque PsO [29].

### Immunogenicity

Treatment-emergent ADAs were reported with secukinumab in < 1% of patients across all studies at week 52. All treatment-emergent ADAs were associated with normal PK [30], and none were associated with loss of secukinumab efficacy or immunogenicity-related AEs.

### Injection site reactions

The EAIR of injection site reactions (high-level term) was 1.2, 1.3, and 0.8 in the PsO, PsA, and AS pools, respectively.

### Incidence of AEs by secukinumab dose

Safety data for any AE, any SAE, serious infections, *Candida* infection, IBD, and MACE for the approved 300 and 150 mg secukinumab dose are shown in Additional file 1: Table S2. The EAIRs were generally comparable, and there was no dose-response relationship observed in terms of safety events with secukinumab treatment within each indication.

### Incidence of AEs year-by-year

**Fig. 2:**
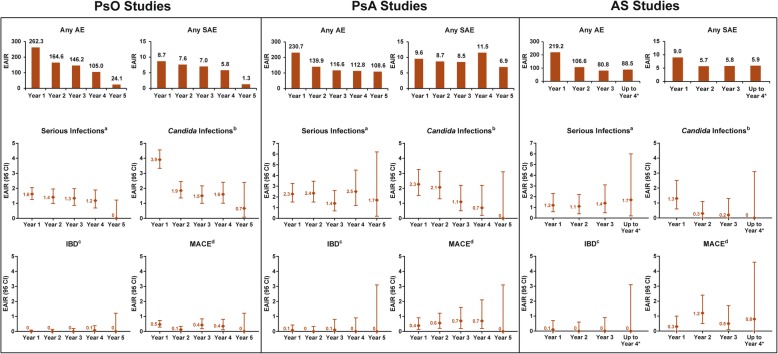
EAIR of AEs year-by-year with secukinumab. Patients included in the analysis: PsO: year 1 (*N* = 5181), year 2 (*N* = 3268), year 3 (*N* = 2246), year 4 (*N* = 1627), and year 5 (*N* = 1210); PsA: year 1 (*N* = 1380), year 2 (*N* = 1183), year 3 (*N* = 948), year 4 (*N* = 587), and year 5 (*N* = 290); AS: year 1 (*N* = 794), year 2 (*N* = 700), year 3 (*N* = 557), and up to year 4 (*N* = 332). *Data shown for patients (*N* = 332) with data beyond the week 156 calendar date (up to 4 years). ^a^Rates are for the system organ class which includes multiple associated PTs. ^b^Rates are for *Candida* infections high-level term which includes multiple associated PTs. ^c^Rates are for PT (IBD PT data are reported for unspecified IBD). ^d^Rates are for the Novartis MedDRA Query term which comprises [1] any MI, any CVA, and [2] all other CV events that are fatal, out of a listing of 2200+ terms. *AE* adverse event, *AS* ankylosing spondylitis, *CV* cardiovascular, *CVA* CV accident, *EAIR* exposure-adjusted incidence rate per 100 patient-years, *IBD* inflammatory bowel disease, *MedDRA* Medical Dictionary for Regulatory Activities, *MACE* major adverse cardiovascular event, *MI* myocardial infarction, *N* number of patients in the analysis, *PsO* psoriasis, *PsA* psoriatic arthritis, *PT* preferred term, *SAE* serious AE

Data on a year-by-year basis for any AE, any SAE, serious infections, *Candida* infection, IBD, and MACE showed no increase with secukinumab treatment over time across studies within each indication (Fig. 2). The EAIR per 100 patient-years for uveitis on a year-by-year basis was as follows: 0.02 (*N* = 5181; year 1), 0.0 (*N* = 3268; year 2), 0.0 (*N* = 2246; year 3), 0.07 (*N* = 1627; year 4), and 0.0 (*N* = 1210; year 5) in PsO clinical trials; 0.2 (*N* = 1380; year 1), 0.0 (*N* = 1183; year 2), 0.0 (*N* = 948; year 3), 0.2 (*N* = 587; year 4), and 0.0 (*N* = 290; year 5) in PsA clinical trials; and 1.1 (*N* = 794; year 1), 1.9 (*N* = 700; year 2), 2.2 (*N* = 557; year 3), and 2.5 (*N* = 332; year 4) in AS clinical trials.

### Post-marketing safety surveillance results

The cumulative post-marketing exposure to secukinumab was estimated to be ~ 96,054 patient-years across the approved indications (Table 4). The exposure-adjusted reporting rates (EARRs) for infections and serious infections were 4.7 and 1.8 per 100 patient-years, respectively. Neutropenia was reported at a rate of 0.07 per 100 patient-years. The reporting rate of hypersensitivity was 2.4 per 100 patient-years. The EARRs for malignancies and MACE were both 0.2 per 100 patient-years, with most assessable cases having multiple confounders, risk factors, or alternative explanations for the events. IBD was reported at a rate of 0.2 per 100 patient-years. The reporting rate for suicidal ideation and behavior was 0.04 per 100 patient-years. Twenty-nine cases of opportunistic infections were reported in the post-marketing data, including 5 cases of TB infection (no reactivation cases), 2 cases of herpes infection, and 17 cases of esophageal candidiasis. There was 1 case of immunogenicity, and no cases of either hepatitis B reactivation or interactions with live vaccines were reported. The safety profile from the PSUR was consistent to that reported in randomized clinical trials (RCTs) with secukinumab.

**Table 4:** Summary of secukinumab post-marketing safety: cumulative and across five PSUR periods

Reporting period	26 Dec 2014–25 June 2015	26 June 2015–25 Dec 2015	26 Dec 2015–25 June 2016	26 June 2016–25 Dec 2016	26 Dec 2016–25 June 2017	Cumulative rate
Exposure	1838	7450	16,871	28,549	41,346	96,054
Infections and infestations/serious infections and infestations						
Cases (*n*)	178/89	495/149	712/232	1136/475	1730/573	4483/1688
EARR (per 100 PY)	9.7/4.8	6.6/2.0	4.2/1.4	4.0/1.7	4.2/1.4	4.7/1.8
Neutropenia						
Cases (*n*)	0	11	12	22	24	66
EARR (per 100 PY)	0	0.2	0.07	0.08	0.06	0.07
Hypersensitivity						
Cases (*n*)	82	293	425	573	752	2293
EARR (per 100 PY)	4.5	3.9	2.5	2.0	1.8	2.4
Malignant or unspecified tumors						
Cases (*n*)	2	15	21	50	76	173
EARR (per 100 PY)	0.1	0.2	0.1	0.2	0.2	0.2
Total IBD						
Cases (*n*)	4	12	37	46	93	195
EARR (per 100 PY)	0.2	0.2	0.2	0.2	0.2	0.2
MACE						
Cases (*n*)	6	15	16	39	58	148
EARR (per 100 PY)	0.3	0.2	0.09	0.1	0.1	0.2
SIB						
Cases (*n*)	1	3	6	8	12	35
EARR (per 100 PY)	0.05	0.04	0.04	0.03	0.03	0.04

Approximation was not done if EARR is less than 0.1

*EARR* exposure-adjusted reporting rates, *IBD* inflammatory bowel disease, *MACE* major adverse cardiovascular events, *PSUR* periodic safety update report, *PY* patient-treatment years, *SIB* suicidal ideation and behavior

## Discussion

In this large safety analysis (*n* = 7355; mean exposure = 16,226.9 patient-years) of 21 clinical trials spanning up to 5 years of treatment for PsO and PsA and up to 4 years of treatment for AS and with safety data from post-marketing surveillance database spanning ~ 96,054 patient-years, secukinumab was associated with a generally low frequency of AEs, with no discernible pattern regarding SAEs across the treatment groups in all indications. The most frequently reported AE was URTI. EAIRs of selected AEs, namely serious infections, *Candida* infections, IBD, and MACE, were consistent with previous reports, with no new safety signals across any indication.

Secukinumab selectively targets IL-17A, a downstream product of Type 17 cells, and leaves the other functions of Th17 cells intact (e.g., the release of IL-22 and TNF) thus limiting the scope for off-target-related effects with secukinumab compared with other biologics. It does not directly influence the Th1 pathway and, thus, is expected to leave Th1-based host immunity largely intact, which may improve the overall safety profile [3, 4, 10]. An increased risk of infections is potentially associated with any immunomodulatory biologic agent [31]. Furthermore, the immune dysregulations underlying severe PsO and spondyloarthritis are also recognized risk factors for an increased potential of developing infections [32]. The use of systemic therapies for PsO seems to further increase this risk of infections [33]. In this analysis, serious infections ranged between an EAIR of 1.2 and 1.9 per 100 patient-years in clinical trials with no clinically meaningful differences in EAIRs across the indications. This incidence of treatment-emergent infections is particularly reassuring and is an important clinical consideration in the management of patients with immune-mediated diseases such as PsO, PsA, and AS.

Neutrophils are important mediators and regulators of innate and adaptive immune responses, and a reduction in peripheral neutrophil counts is a potential effect of immune-modulating agents [34–36], including tofacitinib, brodalumab, and ixekizumab [37, 38]. Over the entire treatment period in this clinical trial safety pool, the EAIRs per 100 patient-years for neutropenia with secukinumab were 0.3, 0.2, and 0.5 in the PsO, PsA, and AS studies, respectively. Also, in the post-marketing safety surveillance, neutropenia was reported as uncommon (EARR of ≥ 1/1000 to < 1/100).

In this large clinical trial safety analysis, the EAIR per 100 patient-years exposures for CD or UC or unspecified IBD was low and ranged between 0.01 and 0.1 in the three cohorts. Discontinuations due to IBD were low with secukinumab (14 [0.19%]). Also, in the post-marketing safety surveillance analysis, the cumulative reporting rate of IBD remained stable at approximately 0.2 reported events per 100 patient-years. Incidence rates per 100 patient-years of CD and UC have been reported in the literature ranging from ~ 0.3 in PsO, 0.1 in PsA, and 0.7 in AS patients [39–41]. Previous exposure to anti-TNF agents and smoking are identified risk factors for IBD exacerbation [42, 43]. In this safety pool, almost one third of all PsA and AS patients were previously exposed to anti-TNF treatments (with an inadequate response or intolerance); previous biologic exposure was also apparent in the PsO cohort, but the rates were lower (15.1%). Each cohort also included a sizeable patient population of current smokers at baseline (~ 30% in PsO and AS, and 19% in PsA). Nevertheless, the overall observed incidence of IBD, including new-onset cases, and discontinuations of secukinumab treatment due to IBD, was uncommon (< 1%). It is important to emphasize that while patients with a previous history of IBD, including CD or UC, could enroll in these trials, the risk of IBD in this cohort could be different from that observed in the real-world, as patients with active IBD were excluded from all clinical trials [40, 44].

Incidences of suicidality-related AEs were low in the PsO and PsA studies, and none were reported in the AS studies. There was no evidence to suggest that treatment with secukinumab increases the risk of suicidality-related AEs beyond background risk in patients with these systemic inflammatory diseases [45, 46].

The incidence of treatment-emergent ADAs was low with secukinumab across all studies evaluated, with no discernible loss of efficacy, unexpected alterations in PK, or association with immunogenicity-related AEs. Considering the reported potential for immunogenicity with secukinumab as compared with other biologic agents [47, 48], this is particularly reassuring and relevant for clinical decision-making.

AE rates calculated on a year-by-year interval basis did not show an increased rate over time for most selected AEs with secukinumab treatment and revealed no new safety signals. EAIR for uveitis was generally low across the three indications. In the AS clinical trials, considering the background rate of uveitis medical history of 17% in the patient population, the decreasing sample size for each subsequent yearly interval may be skewing the EAIRs, given the relative low absolute number of uveitis events reported each year (skewing would be less evident for higher EAIRs). The year-by-year rates should be followed closely as additional long-term exposure data are accumulated. The observed rates of flares and even new-onset uveitis with secukinumab was low and reassuring given the background rate of uveitis in the medical history of this pool (especially in AS patients).

Post-marketing safety data are considered complementary to data from RCTs. Secukinumab was associated with a consistent safety profile in the post-marketing setting across five successive PSUR periods (Dec 26, 2014, to June 25, 2017), with a cumulative post-marketing exposure estimated to be ~ 96,054 patient-years in the approved indications of PsO, PsA, and AS.

Additionally, a recent analysis assessed the outcomes of pregnancies from this safety cohort, which did not find any evidence for increased rates of adverse pregnancy outcomes with secukinumab. However, the analysis was limited by a sizeable amount of missing outcome data and relatively short exposure to secukinumab [49, 50].

Despite reassuring findings, there are limitations to this safety analysis. The conduct of clinical trials is protocol-specified, which may not fully reflect real-world clinical experience. Some studies included in the analysis differ in terms of baseline selection criteria, patient characteristics, and treatment regimens, which is a methodological limitation. However, pooling of data from large clinical trials and post-authorization safety surveillance has enabled a more comprehensive overview of the safety profile of secukinumab. It should be noted that IBD events were not adjudicated in this analysis. The lack of a long-term placebo comparison, due to ethical considerations, limits comparisons. Furthermore, the post-marketing safety surveillance results have not been separated by individual dose regimen or by indication. Rather than using only crude incidence rates, this report included the more robust safety assessment rate by using EAIRs which adjust for potential differences in duration of drug exposure. We believe that factors such as underlying disease activity, co-morbid conditions, and concomitant medications in the patient population are unlikely to have confounded these reported events, but this was not assessed, as it was beyond the scope of the current analyses. The strength of this report is the fact that this integrates safety data from a large patient population pooled over 21 clinical trials across multiple indications, and is complemented with large post-marketing surveillance safety data. The use of EAIRs also enhances the robustness of the results by adjusting for treatment duration. Thus, this report provides valuable evidence on the comprehensive safety profile of secukinumab that should inform clinical decision-making.

### Summary

Secukinumab demonstrated a favorable safety profile over long-term treatment in patients with PsO, PsA, and AS. The safety profile of secukinumab was consistent in these pooled patient populations and with what has been reported earlier for individual studies of secukinumab [12, 13, 15–21, 23, 24, 26, 49]. This long-term (up to 5 years) safety assessment provides a broader understanding of the safety of secukinumab and supports its long-term use in these chronic systemic inflammatory conditions.

## Additional file

Additional file 1: **Table S1.** EAIR for *Candida* infection related preferred terms. **Table S2.** Summary of secukinumab safety by dose. **Table S3.** Summary of studies included in the pooled safety analysis of the entire secukinumab treatment period (from commencement date up to the cut-off date of June 25, 2017). (DOCX 33 kb)
