# Supplementary material for: Long-term safety of secukinumab in patients with moderate-to-severe plaque psoriasis, psoriatic arthritis, and ankylosing spondylitis: integrated pooled clinical trial and post-marketing surveillance data
Source: Arthritis Res Ther. 2019 May 2;21:111. doi: 10.1186/s13075-019-1882-2 (PMC6498580; doi:10.1186/s13075-019-1882-2)
Supplement: Supplementary file 1 — Table S1. EAIR for Candida infection related preferred terms. Table S2. Summary of secukinumab safety by dose. Table S3. Summary of studies included in the pooled safety analysis of the entire secukinumab treatment period (from commencement date up to the cut-off date of June 25, 2017). (DOCX 33 kb) [file 13075_2019_1882_MOESM1_ESM.docx]

**Additional file Table S1. EAIR for *Candida* infection related preferred terms**

| **n (EAIR)** | **PsO Studies** | **PsA Studies** | **AS Studies** |
| --- | --- | --- | --- |
|  | **Any secukinumab N=5181** | **Any secukinumab N=1380** | **Any secukinumab**  **N=794** |
| ***Candida* infections (HLT)** | 221 (2.2) | 57 (1.5) | 13 (0.7) |
| Oral candidiasis | 113 (1.1) | 32 (0.8) | 6 (0.3) |
| Vulvovaginal candidiasis | 49 (0.5) | 12 (0.3) | 2 (0.1) |
| Candida infection | 28 (0.3) | 5 (0.1) | 2 (0.1) |
| Skin *Candida* | 23 (0.2) | 3 (0.1) | 0 |
| Esophageal candidiasis | 10 (0.1) | 4 (0.1) | 1 (0.1) |
| Genital candidiasis | 7 (0.1) | 1 (0.03) | 2 (0.1) |
| Balanitis *Candida* | 6 (0.1) | 2 (0.1) | 0 |
| Gastrointestinal candidiasis | 3 (0.03) | 0 | 0 |
| Oropharyngeal candidiasis | 2 (0.02) | 2 (0.1) | 0 |
| Nail *Candida* | 1 (0.01) | 0 | 0 |
| Nasal candidiasis | 0 | 1 (0.03) | 0 |
| Values are based on the preferred term  Approximation not done for values ≤0.06  Arranged in the descending order of incidence in the PsO group  AS, ankylosing spondylitis; EAIR, exposure adjusted incidence rate per 100 patient-years;  HLT, high-level term; N, number of patients in the analysis; n, number of patients with a response; PsA, psoriatic arthritis; PsO, psoriasis | | | |

**Additional file Table S2. Summary of secukinumab safety by dose**

| **Variable** | **PsO Studies** | | **PsA Studies^** | | **AS Studies** | |
| --- | --- | --- | --- | --- | --- | --- |
|  | **Any**  **Secukinumab**  **300 mg**  **(n=3825)** | **Any**  **Secukinumab**  **150 mg**  **(n=1859)** | **Any**  **Secukinumab**  **300 mg**  **(n=636)** | **Any**  **Secukinumab**  **150 mg**  **(n=857)** | **Any**  **Secukinumab**  **300 mg**  **(n=113)** | **Any**  **Secukinumab**  **150 mg**  **(n=402)** |
| **Total exposure, patient-years** | 6601.4 | 3815.5 | 940.9 | 1913.1 | 196.0 | 961.8 |
| **Exposure (days), mean (SD)** | 630.4  (524.8) | 749.7  (443.2) | 540.4  (408.4) | 815.3  (496.0) | 633.4  (165.24) | 873.8  (367.1) |
| **Death, n (%)** | 6 (0.2) | 3 (0.2) | 0 (0.0) | 8 (0.9) | 0 (0.0) | 1 (0.2) |
| **Discontinuation due to AE, n (%)** | 188 (4.9) | 143 (7.7) | 25 (3.9) | 54 (6.3) | 5 (4.4) | 32 (8.0) |
| **EAIR per 100 patient-years (95% CI)** | | | | | | |
| **Any AE** | 191.1  (184.4,198.0) | 191.6  (182.4, 201.2) | 146.9  (133.2,161.5) | 143.7  (133.1,154.9) | 133.6  (107.7,163.9) | 151.5  (136.0,168.3) |
| **Any serious AE** | 7.0  (6.4, 7.7) | 6.6  (5.8, 7.5) | 7.4  (5.7, 9.4) | 9.0  (7.6, 10.5) | 4.7  (2.2, 9.0) | 5.6  (4.1, 7.3) |
| **Most common AEs^a^** | | | | | | |
| Viral URTI^b^ | 22.2  (20.9, 23.6) | 19.0  (17.5, 20.7) | 12.5  (10.1, 15.2) | 12.6  (10.9, 14.5) | 10.8  (6.5, 16.9) | 10.1  (8.0, 12.5) |
| Headache | 6.6  (5.9, 7.2) | 5.7  (5.0, 6.6) | 3.4  (2.3, 4.8) | 4.1  (3.2, 5.2) | 6.6  (3.4, 11.5) | 5.6  (4.1, 7.4) |
| URTI | 5.2  (4.6, 5.8) | 6.2  (5.4, 7.1) | 9.3  (7.3, 11.6) | 9.0  (7.6, 10.6) | 7.1  (3.8, 12.1) | 4.4  (3.1, 6.0) |
| Diarrhea | 3.7  (3.3, 4.3) | 3.8  (3.2, 4.5) | 3.8  (2.7, 5.4) | 3.8  (3.0, 4.9) | 4.3  (1.9, 8.5) | 5.9  (4.4, 7.7) |
| **Selected AEs with Secukinumab** | | | | | | |
| Serious infection^c^ | 1.3  (1.1, 1.6) | 1.4  (1.1, 1.9) | 2.4  (1.5, 3.6) | 2.0  (1.4, 2.7) | 1.0  (0.1, 3.7) | 0.6  (0.2, 1.4) |
| *Candida* infections^d^ | 2.7  (2.3, 3.1) | 1.4  (1.0, 1.8) | 2.0  (1.2, 3.1) | 1.7  (1.2, 2.4) | 1.0  (0.1, 3.8) | 0.7  (0.3, 1.5) |
| IBD^e^ | 0.0  (0.00, 0.06) | 0.03  (0.00, 0.2) | 0.6  (0.2, 1.4) | 0.2  (0.1, 0.5) | 0.0  (0.0, 1.9) | 0.4  (0.1, 1.1) |
| MACE^f^ | 0.4  (0.2, 0.5) | 0.3  (0.1, 0.5) | 0.1  (0.0, 0.6) | 0.5  (0.2, 0.9) | 0.0  (0.0, 1.9) | 0.4  (0.1, 1.1) |
| ^^^For PsA trials, patients in the secukinumab 150 mg group were allowed to be up-titrated to the 300 mg dose based on clinical assessment, these patients are counted under both “Any secukinumab 300 mg” and “Any secukinumab 150 mg” for the respective period of treatment  ^a^AEs with an EAIR ≥5.0 in either of the three patient pools during the entire treatment period  ^b^Includes cases of common cold (LLT)  ^c^Rates are for system organ class, which includes multiple associated PTs  ^d^Rates are for *Candida* infections high-level term, which includes multiple associated PTs  ^e^Rates reported for unspecified IBD PT  ^f^Rates are for Novartis MedDRA Query term, which includes multiple associated PTs  AE, adverse event; AS, ankylosing spondylitis; CI, confidence interval; IBD, inflammatory bowel syndrome; LLT, low-level term; MACE, major adverse cardiovascular event; PsA, psoriatic arthritis; PsO, psoriasis; PT, preferred term; URTI, upper respiratory tract infections | | | | | | |

**Additional file Table S3. Summary of studies included in the pooled safety analysis of the entire secukinumab treatment period (from commencement date up to the cut-off date of June 25, 2017)**

| **Study name (number)** | **Study identifier** | **Number of patients included** | **Comparator** | **Dose of secukinumab** |
| --- | --- | --- | --- | --- |
| **PsO Studies** | | | | |
| CLEAR  (CAIN457A2317) | NCT02074982 | 335 | Ustekinumab and Placebo | 300 mg qw/q4w SC |
| GESTURE  (CAIN457A2312) | NCT01806597 | 199 | Placebo | 300, 150 mg qw/q4w SC |
| ERASURE  (CAIN457A2302) | NCT01365455 | 702 | Placebo | 300, 150 mg qw/q4w SC |
| JUNCTURE  (CAIN457A2309) | NCT01636687 | 177 | Placebo | 300, 150 mg qw/q4w SC |
| FIXTURE  (CAIN457A2303) | NCT01358578 | 936 | Etanercept and Placebo | 300, 150 mg qw/q4w SC |
| SCULPTURE  (CAIN457A2304) | NCT01406938 | 966 | N/A | 300, 150 mg qw/q4w SC, RAN |
| FEATURE  (CAIN457A2308) | NCT01555125 | 174 | Placebo | 300, 150 mg qw/q4w SC |
| TRANSFIGURE  (CAIN457A2313) | NCT01807520 | 190 | Placebo | 300, 150 mg qw/q4w SC |
| 2PRECISE  (CAIN457A3301) | NCT02008890 | 214 | Placebo | 300, 150 mg qw/q4w SC |
| CARIMA  (CAIN457ADE02) | NCT02559622 | 150 | Placebo | 300, 150 mg qw/q4w SC |
| PSORITUS  (CAIN457ADE03) | NCT02362789 | 130 | Placebo | 300 mg qw SC |
| GAIN  (CAIN457ADE04) | NCT02474069 | 772 | N/A | 300 mg q4w SC |
| PRIME  (CAIN457ADE06) | NCT02474082 | 105 | Fumaric acid esters | 300 mg qw/q4w SC |
| CAIN457JP01 | NCT02547714 | 34 | N/a | 300 mg q4w SC |
| SCALP  (CAIN457AUS01) | NCT02267135 | 97 | Placebo | 300 mg q4w SC |
| **PsA Studies** | | | | |
| FUTURE 1  (CAIN457F2306) | NCT01392326 | 587 | Placebo | 10 mg/kg IV→150, 75 mg q4w SC |
| FUTURE 2  (CAIN457F2312) | NCT01752634 | 387 | Placebo | 300, 150, 75 mg qw/q4w SC |
| FUTURE 3  (CAIN457F2318) | NCT01989468 | 406 | Placebo | 300, 150 mg qw/q4w SC |
| **AS Studies** | | | | |
| MEASURE 1  (CAIN457F2305) | NCT01358175 | 360 | Placebo | 10 mg/kg IV→150, 75 mg q4w SC |
| MEASURE 2  (CAIN457F2310) | NCT01649375 | 211 | Placebo | 150, 75 mg qw/q4w SC |
| MEASURE 3  (CAIN457F2314) | NCT02008916 | 223 | Placebo | 10 mg/kg IV→300, 150 mg q4w SC |
| AS, ankylosing spondylitis; IV, intravenous; PsA, psoriatic arthritis; PsO, psoriasis; qw, once every week; q4w, once every four weeks; RAN, retreatment as needed; SC, subcutaneous | | | | |
